# The Influence of HIV on the Evolution of *Mycobacterium tuberculosis*

**DOI:** 10.1093/molbev/msx107

**Published:** 2017-03-21

**Authors:** Anastasia S. Koch, Daniela Brites, David Stucki, Joanna C. Evans, Ronnett Seldon, Alexa Heekes, Nicola Mulder, Mark Nicol, Tolu Oni, Valerie Mizrahi, Digby F. Warner, Julian Parkhill, Sebastien Gagneux, Darren P. Martin, Robert J. Wilkinson

**Affiliations:** 1 Wellcome Centre for Infectious Disease Research in Africa, Institute of Infectious Disease and Molecular Medicine, and Department of Medicine, University of Cape Town, Cape Town, South Africa; 2 Department of Medical Parasitology and Infection Biology, Swiss Tropical and Public Health Institute, Basel, Switzerland; 3 University of Basel, Basel, Switzerland; 4 Molecular Mycobacteriology Research Unit, Institute of Infectious Disease and Molecular Medicine and Department of Pathology, Faculty of Health Sciences, University of Cape Town, Cape Town, South Africa; 5 Department of Integrative Biomedical Sciences, Institute of Infectious Diseases and Molecular Medicine, University of Cape Town, Cape Town, South Africa; 6 University of Cape Town, and National Health Laboratory Service, Cape Town, South Africa; 7 Division of Public Health Medicine, School of Public Health and Family Medicine, University of Cape Town, Cape Town, South Africa; 8 The Wellcome Trust Sanger Institute, Wellcome Trust Genome Campus, Hinxton, Cambridge, United Kingdom; 9 Division of Computational Biology, Department of Integrated Biology Sciences and Institute of Infectious Disease and Molecular Medicine, Faculty of Health Sciences, University of Cape Town, Cape Town, South Africa; 10 Department of Medicine, Imperial College, London, United Kingdom; 11 Francis Crick Institute, London, United Kingdom

**Keywords:** evolution, *Mycobacterium tuberculosis*, HIV coinfection, natural selection

## Abstract

HIV significantly affects the immunological environment during tuberculosis coinfection, and therefore may influence the selective landscape upon which *M. tuberculosis* evolves. To test this hypothesis whole genome sequences were determined for 169 South African *M. tuberculosis* strains from HIV-1 coinfected and uninfected individuals and analyzed using two Bayesian codon-model based selection analysis approaches: FUBAR which was used to detect persistent positive and negative selection (selection respectively favoring and disfavoring nonsynonymous substitutions); and MEDS which was used to detect episodic directional selection specifically favoring nonsynonymous substitutions within HIV-1 infected individuals. Among the 25,251 polymorphic codon sites analyzed, FUBAR revealed that 189-fold more were detectably evolving under persistent negative selection than were evolving under persistent positive selection. Three specific codon sites within the genes *celA2b*, *katG*, and *cyp138* were identified by MEDS as displaying significant evidence of evolving under directional selection influenced by HIV-1 coinfection. All three genes encode proteins that may indirectly interact with human proteins that, in turn, interact functionally with HIV proteins. Unexpectedly, epitope encoding regions were enriched for sites displaying weak evidence of directional selection influenced by HIV-1. Although the low degree of genetic diversity observed in our *M. tuberculosis* data set means that these results should be interpreted carefully, the effects of HIV-1 on epitope evolution in *M. tuberculosis* may have implications for the design of *M. tuberculosis* vaccines that are intended for use in populations with high HIV-1 infection rates.

## Introduction

Tuberculosis (TB) continues to pose a major public health problem: in 2015, 1.8 million people died of TB, with 22% of these deaths occurring in HIV-1 coinfected individuals (World Health Organization 2016). The synergy between the two diseases is complex: infection with HIV-1 greatly increases the risk of developing TB even before CD4^+^ T-cell counts decrease, and coinfection leads to acceleration of both diseases (Deffur et al. 2013). To develop better approaches to control the syndemic more information is required on how these two pathogens interact with humans.

One tool that might be of utility is comparative genomics. Not long after the first bacterial genome sequence was published (Fleishmann et al. 1995), the genome sequence of *Mycobacterium tuberculosis* was determined (Cole et al. 1998), providing important insights into the biology of this pathogen. Since then, the number of sequenced mycobacterial genomes has rapidly increased owing to decreasing costs of, and greater accessibility to, whole genome sequencing (WGS) technology (Niemann and Supply 2014). Although *M. tuberculosis* is still considered genetically monomorphic compared with other bacterial species (Achtman 2008, 2012), recent WGS data have revealed previously unappreciated degrees of genetic diversity that may have important clinical implications (Gagneux and Small 2007; Sun et al. 2012; Eldholm et al. 2014; Niemann and Supply 2014; Liu et al. 2015).

Evidence suggests that *M. tuberculosis* evolved from a common ancestor in Africa, and spread globally with human migration and trade to give rise to a contemporary phylogeny comprising seven main human-adapted lineages (Hershberg et al. 2008; Comas et al. 2013; Galagan 2014; Brites and Gagneux, 2015). These lineages are associated with particular geographic regions (Hirsh et al. 2004; Hershberg et al. 2008) suggesting sympatric host-pathogen adaptation (Fenner et al. 2013). A population-wide study conducted in Switzerland revealed that HIV disrupts the sympatric relationship between *M. tuberculosis* and particular host populations (Fenner et al. 2013). However, this study was conducted in a region with very low HIV and TB prevalence; transmission patterns might be different in settings where disease burdens are higher and therefore transmission more intense.

In a well-defined, peri-urban community in South Africa, with extremely high HIV-1 and TB prevalence, RFLP typing of strains collected over a 10-year period showed that there was little HIV-specific clustering of *M. tuberculosis* strains (Middelkoop et al. 2009). However, in the mixed clusters, a greater proportion of index cases were HIV uninfected, suggesting that HIV coinfection reduces the likelihood of *M. tuberculosis* transmission (Middelkoop et al. 2015). A population-based study in Malawi, which was one of the first to apply WGS to investigate TB transmission, also found that increased transmission was not associated with HIV coinfection. This study did not, however, assess the directionality of transmission (Guerra-Assunção et al. 2015). Application of epidemiological models to WGS data to study an MDR-TB outbreak in South America, suggest that while HIV coinfection does not lead to increased transmission of TB, HIV coinfected individuals are more susceptible to TB (Eldholm et al. 2016). These studies are consistent with the suggestion that, in settings with high disease burdens, transmission of *M. tuberculosis* from HIV uninfected people to HIV infected people is likely driving the HIV/TB syndemic (Middelkoop et al. 2015).

Host–pathogen interactions have also been investigated using comparative analyses of *M. tuberculosis* WGS data. Studies have revealed that, whereas a small number of antigen-encoding loci in *M. tuberculosis* display variability that appears consistent with selection favoring antibody escape (Coscolla et al. 2015), loci encoding human T-cell epitopes are, counterintuitively, generally conserved (Comas et al. 2010; Coscolla et al. 2015). This suggests either that *M. tuberculosis* requires immune recognition to establish or maintain disease, or that evasion of T-cell mediated immunity is effected by other mechanisms. Since *M. tuberculosis* is able to adapt to the selective pressures imposed by TB chemotherapy (Farhat et al. 2013; Nebenzahl-Guimaraes et al. 2013; Zhang et al. 2013), it is unlikely that antigen conservation reflects an inability of *M. tuberculosis* to efficiently accumulate immune evasion SNPs: epitope conservation may instead fulfil a specific, selectively advantageous, biological function (Coscolla et al. 2015).

A widespread measure used to evaluate the strength and direction of natural selection is the ratio of the rate of nonsynonymous (dN) and synonymous (dS) substitutions in protein coding sequences (Anisimova and Kosiol 2009). Nonsynonymous substitutions result in amino acid changes and are therefore more likely than synonymous substitutions (which do not result in amino acid changes) to influence the functions of encoded proteins. When observed rates of nonsynonymous substitution are disproportionately *higher* than rates of synonymous substitution, it implies that natural selection is likely favoring changes in protein coding sequences that are presumably functionally adaptive: this type of selection is usually referred to as positive selection (Anisimova and Kosiol 2009). Conversely, when observed rates of nonsynonymous substitution are disproportionately *lower* than rates of synonymous substitution, it implies that natural selection is favoring preservation of the protein coding sequences: this is negative or purifying selection (Anisimova and Kosiol 2009).

In the current study, we applied comparative genomics to investigate the impact of HIV-1 coinfection on the evolution of *M. tuberculosis* in a TB and HIV-1 endemic setting. WGS data were generated for 192 *M. tuberculosis* samples isolated from individuals in Khayelitsha, Cape Town, South Africa: a region with one of the highest rates of HIV-1-associated TB in the world (Cox et al. 2014). Phylogenetically informed evaluation of relative synonymous and nonsynonymous substitution rates was used to investigate differences in patterns of natural selection between *M. tuberculosis* strains isolated from HIV-1 coinfected individuals versus uninfected individuals.

## Results

### Phylogeny and Drug Resistance Patterns of *M.tuberculosis* Strains

**Fig. 1.**
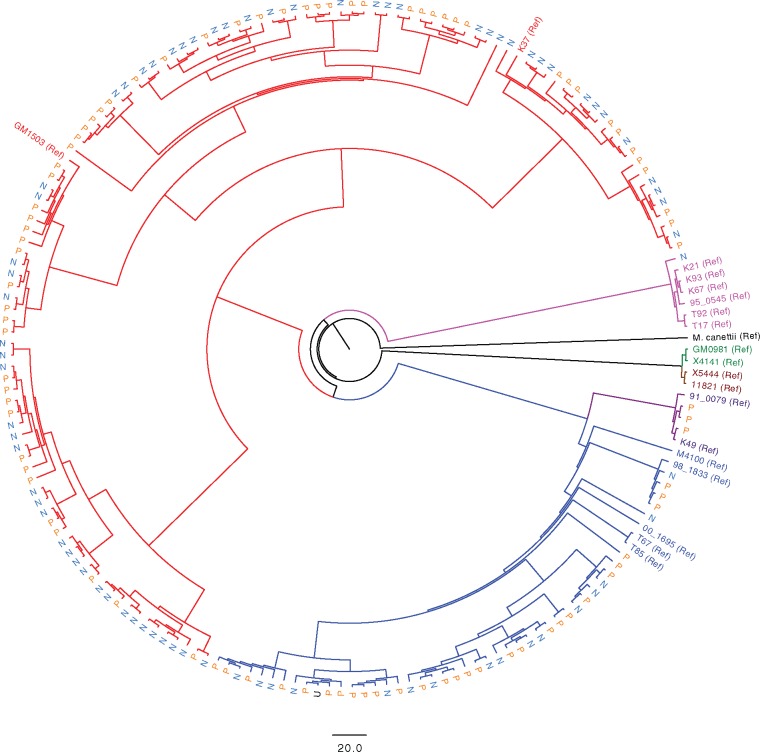
Phylogeny of *M. tuberculosis* strains isolated from HIV-1 coinfected and HIV-1 uninfected individuals. A maximum likelihood phylogenetic tree was constructed using RAxML (Stamatakis 2014). A reference set of strains (annotated as “Ref” on the tree) representative of the known global diversity of *M. tuberculosis* (Comas et al. 2010) was included to provide phylogenetic context for strains isolated in Khayelitsha, South Africa. *M. canettii* is included as an outgroup. Blue and red branches indicate strains belonging to lineage 2 and 4, respectively. Other lineage branches are coloured as in (Comas et al. 2010). Strains isolated from HIV-1 uninfected individuals (N) are indicated in light blue, and those from HIV-1 coinfected individuals (P) in orange. The scale bar indicates SNP differences. The tree with associated bootstrap values is available in the supporting information (supplementary fig. 1, Supplementary Material online).

Of the 192 *M. tuberculosis* isolates that were sequenced, data for four isolates were not included due to under-representation in the multiplexed sequencing library, and data for a further 18 isolates was removed from the analysis due to average sequencing coverage being below 10-fold. One strain isolated from an individual with unknown HIV-1 status was also removed prior to analysis. The mean and median genome sequencing coverage for the 169 strains taken forward for analysis was 80.5 (Standard deviation = 17.9) and 96.2 (Interquartile range = 77.0–90.8) fold, respectively. As expected for this region of South Africa (Hanekom et al. 2013), most of the strains belonged to either lineage 2 (26.0%) or lineage 4 (68.6%; table 1). A small number of strains belonged to lineage 1 (one strain from an HIV-1 uninfected participant) or lineage 3 (three strains, all from HIV-1 coinfected participants). Lineage 2 and lineage 4 strains isolated from HIV-1 coinfected participants made up 16.6% and 33.7% of the sample, and there was no significant association between these *M. tuberculosis* lineages and HIV-1 status. A maximum likelihood phylogenetic tree was constructed using all aligned SNP sites, together with strains representative of global *M. tuberculosis* diversity (Comas et al. 2010) which were included to contextualize the South African sequences within the global *M. tuberculosis* phylogeny (fig. 1 and supplementary fig. 1, Supplementary Material online).

**Table 1:** Characteristics of *Mycobacterium tuberculosis* Samples Included in the Analysis^a^.

	HIV-1 Uninfected *n* (% of Total)	HIV-1 Coinfected *n* (% of Total)	Total	*P* Value^b^
Overall	78 (46.2)	91 (53.8)	169 (100)	
Lineage				
1	1 (0.6)	0 (0)	1 (0.6)	
2	16 (9.4)	28 (16.6)	44 (26.0)	
3	0	3 (1.8)	3 (1.8)	
4	59 (34.9)	57 (33.7)	116 (68.6)	0.114
Mixed infection^c^	2 (1.2)	3 (1.8)	5 (3.0)	
Sex				
Female	28 (16.6)	50 (29.6)	78 (46.2)	
Male	51 (30.2)	40 (23.7)	91 (53.8)	0.009
Median age: years (IQR)				
Female	32.7 (24.8 – 44.0)	32.8 (28.7 – 41.5)		
Male	32.0 (25.9 – 44.5)	38.0 (34.0 – 45.7)		0.824
Year of collection				
2008	20 (11.8)	21 (12.4)	41 (24.3)	
2009	23 (13.6)	16 (9.5)	39 (23.1)	
2010	36 (21.3)	53 (31.4)	89 (52.7)	0.145
RIF resistance	1 (0.6)	7 (4.1)	8 (4.7)	
MDR-TB	0	5 (3.0)	5 (3.0)	
Any resistance	5 (3.0)	8 (4.7)	13 (7.7)	0.249^d^

Note.— RIF, rifampicin; MDR-TB, multidrug resistant TB.

a Although 192 isolates were sequenced, only 170 produced data of high enough quality to be included. The percentage of total sample for each group is indicated in brackets. One lineage 2 strain was isolated from a male with unknown HIV-1 status and was therefore removed from further analysis.

b χ^2^ test between HIV-1 uninfected and HIV-1 coinfected groups.

c Strains comprising mixed infection: lineage 2 and 4 strains (1 HIV-1 coinfected individual, 2 HIV-1 uninfected participants); lineage 2 and 3 strain (1 HIV-1 coinfected individual); lineage 1 and 4 strain (1 HIV-1 coinfected individual).

d Fisher’s Exact Test between HIV-1 uninfected and HIV-1 coinfected groups.

**Table 2:** High Confidence Drug Resistance SNPs Detected in the Data Set^a^.

Lin (HIV-1)^b^	INH *(inhA*)	INH (*katG*)	RIF (*rpoB*)	PZA (*pncA*)	EMB (*embB*)	STR *(rrs*)	*rpoA*	*rpoC*
2 (+)	−15	S315T	S450L	C14R	M306I	513		V483G
2 (+)		S315T	H445D					
2 (+)			S450L	C14R	M306I	513		V483G
2 (+)	−15		S450L			513		
2 (+)			S450L		M306V			
2 (+)	−15		S450L		M306V	516		
4 (−)		S315T						
4 (−)			Q432L					A492V
4 (−)	−15							
4 (−)	−15					513		
4 (−)				C14R				
4 (+)		S315T	S450L					
4 (+)		S315T					T271I	
Total	5	4	8	3	4	6	1	3

a No phenotypic drug susceptibility data was available, therefore only SNPs that are defined as high confidence drug resistance SNPss in the TBDreamDB (Sandgren et al. 2009) are reported. SNPs on each line occur in a single strain.

b Indicates the lineage of the strain and whether the strain was isolated from an HIV-1 coinfected or HIV-1 uninfected individual.

The genomic data were examined for drug resistance conferring SNPs. Although 341 SNPs were observed in candidate drug-resistance genes, phenotypic susceptibility results were not available to confirm genotypically inferred resistance. Therefore, only SNPs that were defined as “high confidence” resistance SNPs in the TBDreamDB (Sandgren et al. 2009) were reported as likely drug-resistance SNPs (table 2). By this criterion, 4.7% (*n* = 8) of the sequenced strains were likely to be rifampicin (RIF) resistant, which is consistent with previous reports for Khayelitsha (Cox et al. 2010). Of the eight RIF-resistant strains, three were also likely to be resistant to isoniazid (INH); therefore, the individuals infected with these strains probably had multidrug resistant TB (MDR-TB). Although additional resistance SNPs associated with ethambutol (*n* = 4), pyrazinamide (*n* = 3), and streptomycin (*n* = 5) were observed, no fluoroquinolone resistance SNPs were observed (even when entire *gyrA* and *gyrB* genes were considered), thus none of the individuals had preextensively drug resistant TB (pre-XDR-TB).

### Patterns of Selection at Individual Sites within the *M.tuberculosis* Genome

Two approaches were applied to evaluate natural selection within the *M. tuberculosis* coding sequences. First, the FUBAR method (Murrell et al. 2013), which draws information about selection from all branches of a phylogenetic tree, was used to investigate overall patterns of positive and negative selection in the *M. tuberculosis* genome without differentiating sequences according to HIV-1 infection status. Second, the MEDS method (Murrell et al. 2012), which queries changes in selection along specific branches thought to transition between two environmental variables (often occurring at terminal branches), was applied to identify signals of directional positive selection along specific branches of the phylogeny marking likely transitions of *M. tuberculosis* from HIV-1 uninfected individuals to HIV-1 coinfected individuals: signals of selection in *M. tuberculosis* that might be indicative of adaptation to HIV-1 coinfection.

Of 28,857 SNPs within the analyzed genome sequences, 25,251 (87.5%) occurred in protein coding regions. The codon sites containing each of these SNPs were assembled into codon alignments (38,544 nucleotides long for the sense alignment and 36,498 nucleotides long for the antisense alignment), which were then used to determine codon site-specific dN–dS values with FUBAR. FUBAR estimates the distribution of dN and dS substitutions across an analyzed coding region, and applies a Bayesian Markov chain Monte Carlo (MCMC) approach to determine which dN and dS combination each site is most likely to have (Murrell et al. 2013).

Short-read sequencing data may be unreliable for genomic regions that contain repeat sequences. Approximately, 10% of the *M. tuberculosis* genome contains repetitive regions that are can cause difficulty during mapping of short-read sequencing data (Galagan 2014). These regions were removed during genome assembly and variant calling, prior to alignment and phylogenetic analysis (supplementary table 4, Supplementary Material online). The number of strains for which a nucleotide could not be called at a site is indicated in supplementary table 1, Supplementary Material online. FUBAR results were further filtered to exclude sites occurring in recently described regions for which short-read sequencing data might also be unreliable (Coscolla et al. 2015). After this filtering step, data for 23,860 codon sites remained.

This analysis confirmed that the codons containing the most frequently occurring drug-resistance SNPs in this data set (S531T in *katG*, dN–dS = 0.29, and S450L in *rpoB*, dN–dS = 0.65, supplementary table 1, Supplementary Material online) were evolving under positive selection (i.e., selection favoring changes away from a drug-sensitive state). This result provided important validation that, in the context of our data set, FUBAR could detect expected signals of positive selection.

**Fig. 2.**
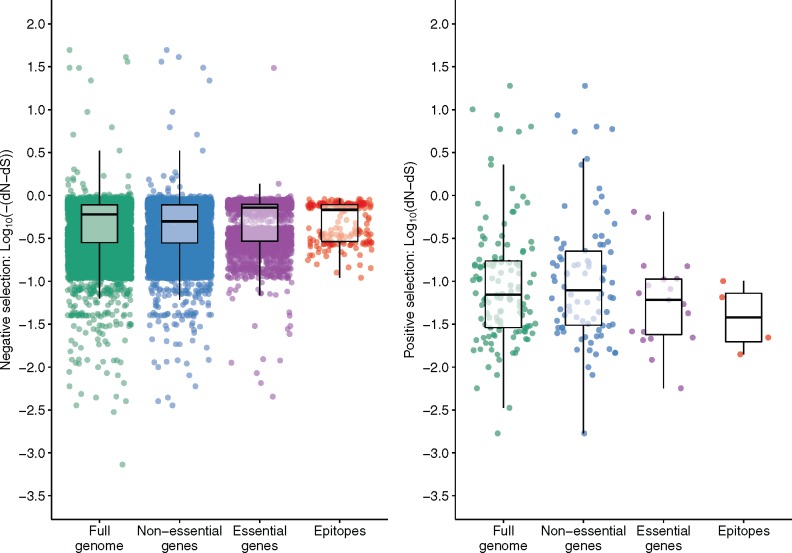
Strengths of natural selection differ for different functional gene categories in *M. tuberculosis*. Estimates at 23,860 codon sites within the analyzed *M. tuberculosis* genomes were evaluated for nonsynonymous (dN) and synonymous substitution rates (dS) without considering HIV-1 status using the FUBAR method (Murrell et al. 2013). Analyzed codon sites were classified into essential, nonessential and epitope encoding regions (Sassetti et al. 2003). The left panel shows codons that are under negative selection (dN–dS < 0). Each dot represents the log10 of the −dN–dS values reported by the FUBAR analysis. The right panel shows codons under positive selection dN–dS>0, with each dot representing the log10 of dN–dS values. The boxplots represent the interquartile range (IQR), the median and the highest and lowest values within 1.5 times the IQR and outliers are captured by the scatterplot. Wilcoxon rank sum tests were performed by comparing the distribution of dN–dS values for all individual codons in each category: essential versus nonessential (*P* < 0.001); essential versus epitope (*P* = 0.192); nonessential versus epitope (*P* = 0.336).

Interestingly, a site in *mce1F* (G418G, dN–dS = −49.65, supplementary table 1, Supplementary Material online), part of an operon encoding an ABC transporter implicated in host–pathogen interactions (Shimono et al. 2003) showed some of the highest degrees of negative selection (i.e., selection favoring conservation in amino acid state). Conversely one of the codon sites inferred to be under the strongest degree of positive selection was located in Rv0988 (L191V, dN–dS = 18.95, supplementary table 1, Supplementary Material online), a gene also predicted to be part of an operon encoding an ABC transporter (Rosas-Magallanes 2006), whose product is highly expressed *in vivo* (Talaat et al. 2004). A cytochrome P450 enzyme (*cyp138*, P114S, dN–dS = 8.62) and a conserved hypothetical protein predicted to be associated with the cell membrane (Rv1417, S102P, dN–dS = 10.09) were two other codon sites detected by FUBAR to be evolving under positive selection. Importantly, of the total 23,860 codons analyzed, 0.26% contained three or more inferred amino acid changes providing enough diversity to enable FUBAR to estimate dN and dS values for at least 62 sites within the genome (supplementary table 1 and fig. 2, Supplementary Material online). Annotated phylogenetic trees showing the position of nonsynonymous substitutions on the phylogeny underlying the positive selection signals at these sites are shown in supplementary fig. 3, Supplementary Material online.

Genome sites analyzed for evidence of selection were classified according to whether they fell in essential genes, nonessential genes (Sassetti et al. 2003), or epitope encoding regions (fig. 2). Previous studies (Comas et al. 2010; Coscolla et al. 2015) have suggested that epitopes are more highly conserved than essential and nonessential genes. In this study, epitopes had a lower median dN–dS value (−0.62) than nonessential genes (−0.48) (table 3) however essential genes had an even lower median dN–dS (−0.72) than epitopes (−0.62). Differences in the distribution of dN–dS values between essential genes and epitopes (*P* = 0.192, Wilcoxon rank sum test) or between nonessential genes and epitopes (*P* = 0.336) were not significant.

**Table 3:** Distribution of Site-Specific dN–dS Values Across Different Gene Categories.

Gene Category	Median	Range
Full genome	−0.60	−49.65 to 18.95
Essential genes	−0.48	−30.68 to 0.66
Non-essential genes	−0.72	−49.65 to 18.95
Epitopes	−0.62	−0.91 to 0.10

### Specific Codon Sites with Strong Evidence for Directional Selection Attributable to *M.tuberculosis* HIV-1 Coinfection

The MEDS method (Murrell et al. 2012) was used to identify instances of positive selection that might specifically be influenced by HIV-1 coinfection. Rather than reporting an accurate dN–dS value at each site, the MEDS method reports the probability of a different encoded amino acid state being favored in the foreground sequences (in this case *M. tuberculosis* sequences from HIV-1 coinfected individuals) than that which is favored in the background sequences (in this case *M. tuberculosis* from HIV-1 uninfected individuals) (Murrell et al. 2012). Based on maximum likelihood codon and nucleotide substitution frequency estimates, the given tree topology and the foreground/background sequence designations, MEDS will identify codon sites as potentially evolving under directional positive selection when: 1) these sites display a significant tendency to only incur nonsynonymous substitutions along branches marking transitions from background to foreground sequences and 2) the nonsynonymous substitutions at these sites involve nucleotide or amino acid changes that, relative to those observed in the remainder of the data set, are unusually rare.

**Fig. 3:**
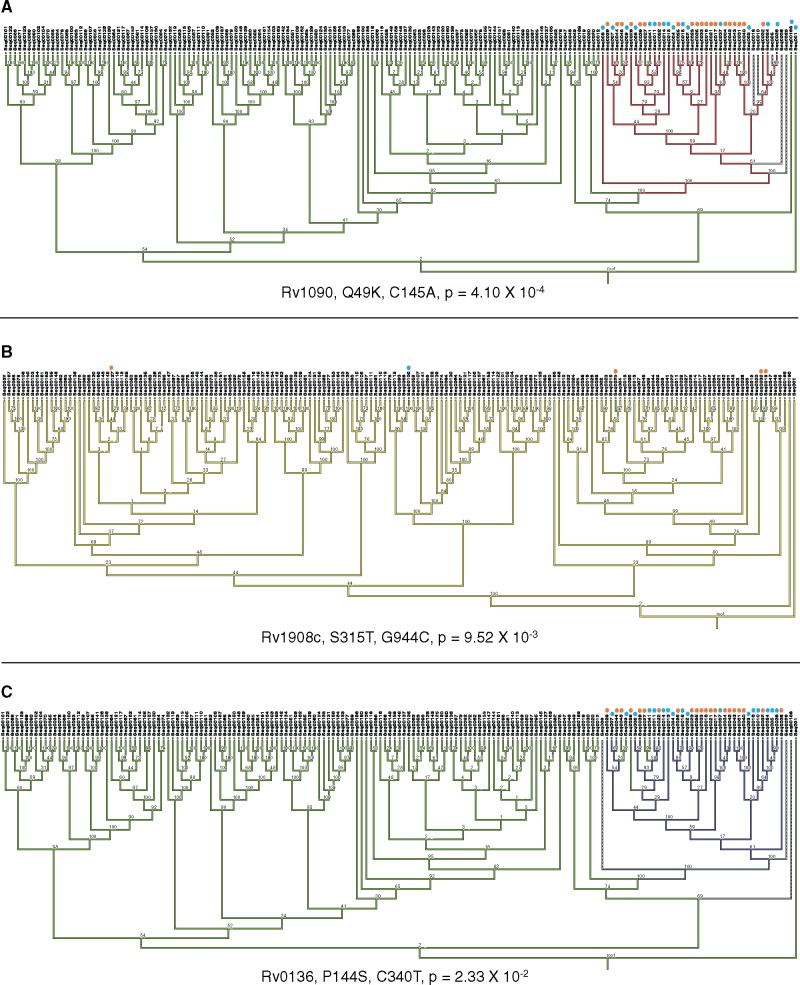
Phylogenetic mapping of *M. tuberculosis* codon sites with highly significant *P* values for evidence of HIV-1 associated directional selection. MESQUITE (Maddison and Maddison, 2017) was applied to map the phylogenetic position of codon sites with intermediate to low MEDS associated *P* values. For each site the amino acid change, the nucleotide change and the degree of Bonferroni corrected *P* value support is indicated. *M. tuberculosis* strains with the mutation isolated from HIV-1 coinfected individuals are indicated with an orange dot, and those from HIV-1 uninfected individuals a blue dot. Phylogenetic trees were generated in RAxML (Stamatakis 2014) and bootstrap values from 1000 replicates are annotated on nodes. (*A*) The site with the strongest evidence of directional selection in HIV-1 coinfected individuals is shown. Branches that contain the ancestral cytosine are indicated in green, while those that contain adenine are shown in red, dashed branches indicated branches for which there was no call at that site for that strain. (*B*) Phylogenetic mapping of the S513T mutation in the *katG* gene (Rv1908c). This substitution involves a single nucleotide substitution from guanine (in yellow) to cytosine (in green). (*C*) The third codon site very strong evidence of HIV-1 associated directional selection occurs in *cyp138* (Rv0136). The nucleotide change at this site is from cytosine (in green) to thymine (in purple).

After filtering for sites that may occur in regions for which short-read sequencing data may be unreliable, at a significance threshold of *P* < 0.05, a total of 640 sites (supplementary table 2, Supplementary Material online) showed evidence of possible directional positive selection across phylogenetic tree branches separating *M. tuberculosis* strains sampled from HIV-1 uninfected (in the background) and HIV-1 coinfected (in the foreground) individuals. It is important to note that the *P* values associated with these sites were not multiple testing corrected and, therefore, that a large proportion of these sites could be false positives. After applying a Bonferroni multiple testing correction only three codon sites, in the genes *celA2b* (Rv1090), *katG* (Rv1908c), and *cyp138* (Rv0136), remained significant (table 4). Importantly, phylogenetic substitution patterns for these three sites confirmed the occurrence of nonsynonymous substitutions along terminal tree branches with *M. tuberculosis* isolates from HIV-1 infected persons at their tips: an observation that is consistent with the action of HIV coinfection influenced directional selection at these sites (fig. 3).

**Table 4:** Codons with Highly Significant Evidence of HIV-1-Influenced Directional Selection.

Gene	Amino Acid Substitution	Nucleotide Substitution	Bonferroni Corrected MEDS *P* Value	Predicted Function According to Tuberculist
*celA2b* (Rv1090)	Q49K	C145A	4.10 × 10^−4^	Endoglucanase
*katG* (Rv1908c)	S315T	G944C	9.52 × 10^−3^	Catalase-peroxidase
*cyp138* (Rv0136)	P114S	C340T	2.33 × 10^−2^	Cytochrome P450

**Fig. 4.**
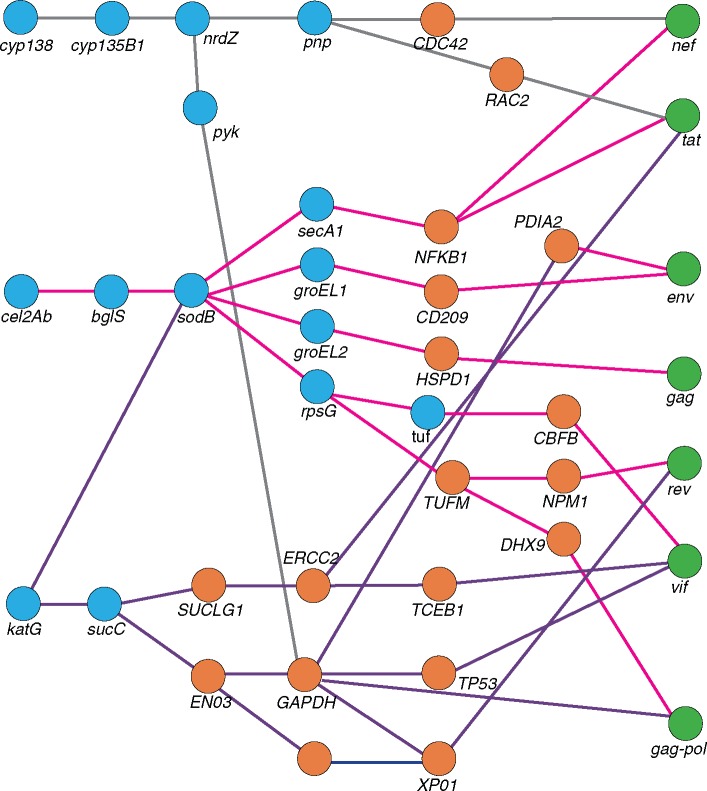
Inferred functional interactions between *M. tuberculosis* genes with codons under HIV-1 associated directional selection and human and HIV-1 genes. A functional interaction analysis was conducted to investigate the biological significance of *M. tuberculosis* genes with evidence for directional selection influenced by HIV-1 coinfection. Data for intraspecies human–human, *M. tuberculosis–M. tuberculosis* interactions was retrieved from STRING (Szklarczyk et al. 2015) and REACTOME (Fabregat et al. 2016; Croft et al. 2014), with a cutoff confidence score of 0.7. Human–HIV-1 interactions were derived from the HIV-1 Human Protein Interaction Database (Fu et al. 2009) and human–*M. tuberculosis* interactions were retried from previously generated data (Rapanoel et al. 2013 and Huo et al. 2015). For both interspecies interactions, data was only included if previously reported in the literature. Blue circles represent genes from *M. tuberculosis*, orange circles from humans, and green circles from HIV-1.

To investigate the potential biological significance of HIV-1 influenced directional selection at codon sites in *celA2b*, *katG*, and *cyp138*, we determined the shortest pathways between these genes and those of HIV-1 in an inferred three-way *M. tuberculosis*–human–HIV functional protein interaction network. The nature of this network precludes direct *M. tuberculosis*–HIV interactions such that all interactions between *M. tuberculosis* and HIV must occur via an intermediary human gene. With stringent filtering parameters, the interactions in the shortest paths with the highest scores between *M. tuberculosis* genes with evidence of HIV-1 influenced selection (table 4) and HIV-1 genes is shown in figure 4. The shortest distance (path length of 4) occurred between *katG* and HIV-1 genes *nef*, *tat*, *env*, *gag*, and *gag-pol*. The immunologically important human genes forming functional bridges between HIV-1 and *M. tuberculosis* proteins include specific intercellular adhesion molecule-3 grabbing nonintegrin (DC-SIGN) receptor on dendritic cells (between *M. tuberculosis celA2b* and *katG*, and HIV-1 *env*) and NF-κβ (between *celA2b* and HIV-1 *tat* and *nef*).

### *M.* *t* *uberculosis* Gene Categories with an Enrichment of Codon Sites Showing Evidence of Directional Selection Influenced HIV-1 Coinfection

Although the MEDS analysis identified only three sites with Bonferroni corrected *P* values that were < 0.05, it is plausible that the remaining pool of 637 sites identified as evolving in a way that is consistent with directional selection that is influenced by HIV-1 coinfection (i.e., with MEDS *P* values < 0.05 prior to Bonferroni multiple testing correction), is enriched for sites that are actually evolving under HIV-1 influenced directional selection. However, as MEDS inferred *P* values increase (i.e., evidence for directional positive selection decreases) at individual sites, there is a decrease in the numbers of nonsynonymous substitutions at these sites that map to tree branches that separate samples from HIV-1 coinfected and HIV-1 uninfected individuals (supplementary fig. 4, Supplementary Material online), and it is therefore likely that a proportion of these 640 sites are false positives. For this reason, we treated the 640 sites with Bonferroni uncorrected MEDS *P* values of < 0.05 as a pool of sites that, rather than representing a list of all directionally evolving sites, are enriched for sites evolving under HIV-1 influenced directional selection relative to the other 23,220 polymorphic sites in our data set that were not reported by the MEDS method as significant.

We analyzed this pool of 640 sites by first categorizing them according to whether they occurred in essential genes, nonessential genes or epitope encoding regions (supplementary table 2, Supplementary Material online), and then testing whether particular site categories were over-represented in the 640 site pool relative to what would be expected if sites in the 640 site pool were randomly distributed throughout the coding region of the *M. tuberculosis* genome.

**Fig. 5.**
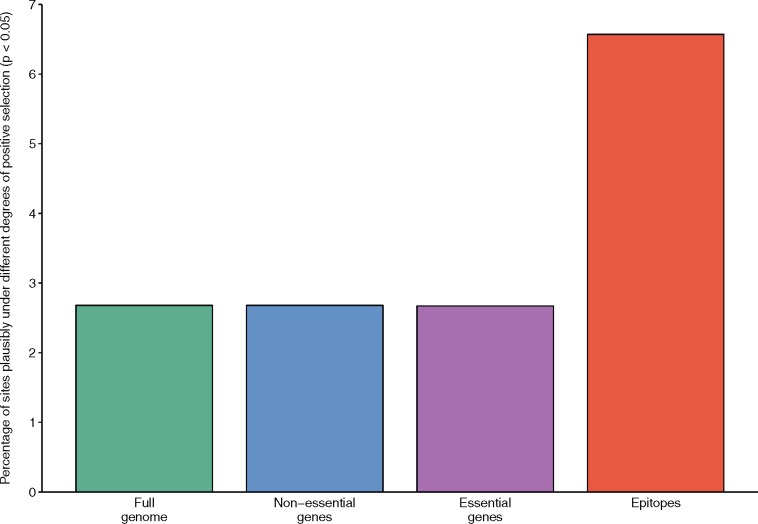
Coding sites in epitope encoding regions are enriched for evidence of directional selection influenced by HIV-1 coinfection. Episodic directional selection in *M. tuberculosis* strains isolated from HIV-1 uninfected and HIV-1 coinfected individuals was evaluated using the MEDS method (Murrell et al. 2012). Sites that showed evidence of directional selection associated with phylogenetic branches separating HIV-1 uninfected and HIV-1 coinfected individuals (i.e., with an associated MEDS *P* value < 0.05, without Bonferroni multiple testing correction) were assigned to essential, nonessential and epitope encoding gene categories (Sassetti et al. 2003). The percentage of sites falling in each category is indicated by the bars. The site counts used to calculate these percentages can be seen in supplementary table 3, Supplementary Material online.

The 640-site pool contained a greater number of epitope sites than expected (fig. 5). Specifically, while only 198 of the codons that fall within known *M. tuberculosis* epitopes contained SNPs in our data set, 13 of these SNP containing codons were among the 640 sites that were identified by MEDS as displaying nucleotide substitutions that are consistent with HIV-1 coinfection influenced directional selection. This number is significantly higher than the number of sites within epitopes that would be expected if the 640 codons under consideration were randomly scattered throughout the genome (*P* = 0.001; 2-tailed Fisher’s exact test; fig. 5). This number was also notably higher than when the MEDS analysis was rerun with *M. tuberculosis* sequences from HIV-1 uninfected individuals in the foreground and those from infected individuals in the background (supplementary table 3, Supplementary Material online). In this control analysis no significant enrichment of sites within epitopes was found amongst the pool of sites identified as potentially evolving under directional selection (*P* = 0.09, 2-tailed Fishers exact test; supplementary table 3, Supplementary Material online).

We next used a permutation test to more rigorously determine whether the observed enrichment of potentially directionally evolving sites in epitopes could be accounted for by chance. In this test, the HIV-1 infection status of *M. tuberculosis* sequences were randomly reassigned to produce 100 permuted data sets each of which were reanalyzed with MEDS. None of these 100 permuted data sets yielded evidence of as many directionally evolving sites in epitopes as were found in the real data set: a result equivalent to a permutation *P* value <0.01 that HIV-1 infection status, when randomly assigned to sequences, was not influenced by directional selection in epitope sequences (supplementary table 3, Supplementary Material online).

## Discussion

This study applied WGS and codon-site molecular evolution models to investigate the potential influence of HIV-1 coinfection on the diversity and evolution of *M. tuberculosis* strains in Khayelitsha, South Africa; a setting with elevated rates of HIV-1-associated TB (Cox et al. 2014). To investigate the evolution of *M. tuberculosis* strains in this setting, two approaches were taken. The first was to apply the FUBAR method to evaluate patterns of positive and negative selection at individual codon sites within the *M. tuberculosis* genome without considering differential evolution of *M. tuberculosis* in HIV-1 coinfected individuals. The second was to apply the MEDS method to detect episodic directional selection that may be influenced by HIV-1 coinfection.

Some studies (Rocha et al. 2006; Kryazhimskiy and Plotkin 2008; Mugal et al. 2014) have suggested that when analyzing sequences that are too closely related, dN/dS estimates may not accurately reflect degrees of natural selection acting upon the sequences. The FUBAR analysis provides evidence that sufficient time has passed since the most recent common ancestor of the sequences analyzed for selection to have left a detectable imprint. Specifically, this legitimacy of using the FUBAR approach is supported by the fact that it enabled 1) the detection of almost all of the same positively evolving *M. tuberculosis* codon sites that were identified in another study applying similar codon-model based approaches (Osório et al. 2013), and 2) the identification of some drug resistance associated SNPs as evolving under positive selection.

An advantage of using FUBAR to detect the action of natural selection is that it simultaneously quantifies magnitude of both positive and negative selection at individual codon sites. Among the codon sites that contained SNPs in the analyzed *M. tuberculosis* genomes, 99.47% were detectably evolving under negative selection (*n* = 23,763) while only 0.52% were detectably evolving under positive selection (*n* = 125) (fig. 2), which is in line with findings previously reported in other studies (Pepperell et al. 2013).

A key aim of our study was to determine if HIV-1 coinfection, which represents a relatively recent addition to the adaptive landscape upon which *M. tuberculosis* evolves (Warner et al. 2015), has had a detectable influence on *M. tuberculosis* evolution. To do this, each SNP containing codon site was evaluated using the MEDS method to determine whether selection pressures acting on these codon sites could differ between HIV-1 coinfected and HIV-1 uninfected individuals (Murrell et al. 2012). In a recent study, Eldholm et al. (2016) investigated the impact of HIV on the transmission and development of drug resistance in *M. tuberculosis* (Eldholm et al. 2016). The results of that study suggest that HIV has little impact on the transmission of *M. tuberculosis*, and that drug resistance does not develop faster in patients coinfected with HIV. However, our study is the first to conduct a genome-wide site-by-site investigation of the impact of HIV-1 on natural selection in *M. tuberculosis*.

The first of three codons with associated Bonferroni corrected MEDS *P* values indicative of HIV-1 influenced directional selection (table 4) occurs in *celA2b*, a nonessential gene which encodes a functional endoglucanase (Medie et al. 2011). The second occurred in *katG*, which encodes a catalase-peroxidase enzyme that is important for *M. tuberculosis* virulence and is known for its association with INH resistance (Pym et al. 2002; Yu et al. 2003; Wengenack et al. 2004), also had significant evidence of HIV-1 influenced directional selection. The third codon occurred in *cyp138*, a cytochrome P450 encoding gene (Hudson et al. 2012).

The biological significance of these codons during *M. tuberculosis*/HIV-1 coinfection was not obvious, therefore we determined whether there were either direct or indirect known functional interactions between the proteins encoded by these genes and human proteins that are known to interact with HIV-1 proteins (fig. 4). Both CelA2b and KatG are involved in an interaction pathway that, via SodB and ultimately GroEL1, leads to DC-SIGN, an important receptor for both HIV-1 and *M. tuberculosis* on DC (van Kooyk et al. 2003). Although these interactions may begin to explain why these *M. tuberculosis* sites display evidence of directional selection influenced by HIV-1 coinfection, these findings would benefit further investigation in experimental systems.

The results of the MEDS analysis also indicated differences in directional selection influenced by HIV-1 coinfection at codon sites falling in different gene categories. The proportion of sites with associated MEDS *P* values of < 0.05 was higher in the epitope category (fig. 5) than in either the essential or nonessential gene categories. Importantly, the enrichment in epitopes was not observed when the MEDS analysis was rerun with either HIV-positive infection status as the background state (fig. 5 and supplementary table 3, Supplementary Material online) or following 100 randomizations of the HIV-infection statuses of the individuals from which the *M. tuberculosis* isolates were isolated. This indicates that the observed enrichment in epitopes of codons potentially evolving under directional selection influenced by HIV-1 coinfection is unlikely to be an artifact of the underlying assumptions of the evolutionary model applied.

Nevertheless, 92.66% of the 640 sites we selected as being enriched for elevated signals of potential directional selection influenced by HIV-1 coinfection had MEDS derived *P* values ranging from 0.01 to 0.05 (i.e., these sites individually displayed evidence of directional selection). Only a few nonsynonymous mutations were observed at each of these sites (supplementary fig. 4, Supplementary Material online), and it is therefore important to reiterate that a proportion of these 640 sites are likely false positives. Therefore, while we are confident that the signal of elevated HIV-1 influenced directional selection at epitope sites that we detected by collectively considering all of these “marginally significant” sites is present in the particular *M. tuberculosis* data set that we have examined, it would be desirable to validate findings in an independent data set. This is particularly so because our study is the first which has applied methods such as MEDS to analyzing data sets with a degree of genetic diversity that is as low as that found in our *M. tuberculosis* data set.

Besides those already mentioned there are several additional caveats associated with the analysis performed here. The duration of the HIV-1 and *M. tuberculosis* infections prior to strain isolation could not be determined. This is important: uncertainty about when during the evolutionary history of a group of coding sequences a particular environmental variable changed (in this case HIV-1 status), undermines the power of methods such as MEDS to attribute specific encoded amino acid changes to specific changes in the environmental variable. Nevertheless, simulated and empirical data set analyses have indicated that MEDS is reasonably conservative and, provided sequences assigned to the two groups are mixed in the phylogeny (as is the case here where there are not large clusters of exclusively HIV-1 coinfected or HIV-1 uninfected sequences), it is robust to uncertainty regarding the precise positions along an analyzed phylogeny where the environmental variable switched states (Murrell et al. 2012).

The second additional caveat is that the genetic diversity of HIV-1 infection may be important when considering the global significance of these results. Several studies have suggested that variation in rates of disease progression (Touloumi et al. 2013; Mlisana et al. 2014), transmissibility (Abraha et al. 2009), and decline in CD4^+^ T-cell numbers are associated with the genotype of HIV-1 such that, for example, viruses belonging to one subtype might in general be more transmissible than those in another subtype (Ariën et al. 2007). This study was conducted in Khayelitsha, Cape Town, South Africa, where HIV-1 subtype C accounts for >99% of all HIV-1 infections (Jacobs et al. 2006). In cohorts with different rates of HIV-1 associated TB or where different HIV-1 subtype(s) predominate, the coevolutionary dynamics of HIV-1 and *M. tuberculosis* may be different. This provides further motivation to repeat this analysis on different cohorts.

These caveats notwithstanding, we have effectively used MEDS and FUBAR as computational methods to uncover new hypotheses that could potentially be subject to downstream experimentation. The application here of sophisticated codon evolution models to detect HIV-1 influenced selection in *M. tuberculosis* paves the way both for future investigations of larger, more carefully defined, *M. tuberculosis* WGS data sets to validate the findings, and for studies seeking to determine whether there are specific underlying molecular causes of differential selection pressures on *M. tuberculosis* in HIV-1 coinfected and uninfected individuals.

## Materials and Methods

### Sample Selection Criteria

One-hundred and ninety two *M. tuberculosis* isolates were selected from three separate clinical studies performed between 2008 and 2010 (Berry et al. 2010; Boehme et al. 2010; Kaforou et al. 2013). The University of Cape Town Faculty of Health Sciences Human Research Ethics Committee provided ethical approval (HREC 012/2007). All studies were conducted at the uBuntu clinic in Site B, Khayelitsha, a peri-urban township 30 km outside of Cape Town, South Africa. In 2010, the study setting had an estimated adult HIV-1 prevalence of 33% and a TB incidence of 1,500/100,000 (Cox et al. 2014). *M. tuberculosis* isolates were selected randomly from stored Mycobacterial Growth Indicator Tube (MGIT) (Beckton Dickinson, USA) samples and were included in this study if the HIV-1 status of the *M. tuberculosis*-infected participant was known. All *M. tuberculosis* samples were isolated from persons who had not yet received antiretroviral therapy.

### Strain Culture and DNA Extraction

*M. tuberculosis* isolates were subcultured from glycerol stocks (originally made from positive MGITs) onto LJ slopes. Upon confluent growth (after 4–6 weeks), DNA was extracted from a pellet of cells. Extractions were conducted according to (van Helden et al. 2001), except that 710 µl of a 5M NaCl solution and 820 µl of a 10% CTAB solution were added to samples after proteinase K treatment and samples were incubated for 10 min at 65 °C before continuing the extraction.

### Whole Genome Sequencing

Samples were sequenced at the Wellcome Trust Sanger Institute using 100bp paired-end libraries on an Illumina HiSeq 2000 instrument. Libraries were prepared as previously described (Quail et al. 2008), except that KAPA HiFi polymerase was used instead of Phusion for PCR-amplification steps (Quail et al. 2012). Ninety-six individually tagged libraries were sequenced in a single lane according to the manufacturer’s instructions.

### Genome Assembly and Detection of Variants

BWA v0.6.2 (Li and Durbin 2009) was used to map paired-end reads to a previously reconstructed ancestor of *M. tuberculosis* (Comas et al. 2010). SAMtools v0.1.18 (Li et al. 2009) was used to call variant positions. Strains that had less than 10-fold average coverage were excluded from further analysis. Sites that had Phred scores lower than 20 or coverage below 10-fold were also removed. Annovar (Wang et al. 2010), using the H37Rv database, was used to annotate variant positions. Indels and repetitive regions and those related to mobile genetic elements (supplementary table 4, Supplementary Material online) were removed from all sequences prior to further analysis. SNPs in individual sequences that were supported by fewer than 95% of reads were also removed. Reads for *M. tuberculosis* lineage reference strains (Comas et al. 2010) were downloaded from the NCBI Sequence Read Archive. These strains were sequenced using single-end technology, and were therefore mapped using single-end parameters on BWA, and analyzed further with the same quality thresholds as those described above.

### Drug Resistance and Lineage Typing

Lineage specific SNPs were extracted for each strain, and samples for which mixed infection was evident were removed from further analyses. SNPs in all candidate drug-resistance associated genes described in the TBDReamDB (Sandgren et al. 2009) were extracted. Given that phenotypic susceptibility tests for the data set were not available, only SNPs annotated as high confidence on the TBDReamDB (Sandgren et al. 2009) were reported. All observed SNPs in *rpoA* and *rpoC* were also reported.

### Phylogenetic Tree

MAFFT v7 (Katoh and Standley 2013) was used to align sequences with the FFT-NS-i refinement parameter and a PAM (*k* = 2) scoring matrix. RAxML v8.1.20 (Stamatakis 2014) was used to construct a phylogenetic tree using the GRTCAT model with 1,000 bootstrap iterations.

### Evolutionary Modeling and Detection of Selection

Prior to evaluating signals of natural selection, the *M. tuberculosis* WGS sequence alignment was tested for evidence of recombination using RDP4.56 (Martin et al. 2015) with default settings. To assemble codon alignments individual SNPs were mapped to codons in the H37Rv annotation, following which codons on the sense and antisense strands were extracted and assembled into codon alignments. Since SNPs occurred in genes located in both the sense and antisense orientations of the genome, two codon alignments were generated. These two alignments were separately analyzed with the FUBAR (Murrell et al. 2013) and MEDS (Murrell et al. 2012) methods implemented in HYPHY (Kosakovsky Pond et al. 2005). For FUBAR, the universal genetic code was used (as no specific bacterial code is available for the analysis); parameters changed from default settings were: (1) 10 Markov chain Monte Carlo (MCMC) chains were used in phase 3, (2) the length of each chain was increased to 1,000,000, (3) the first 50,000 links of each chain were discarded as burn-in, (4) 300 postburn-in samples were drawn from each chain, and (5) the concentration of the Dirichlet prior was set to 0.5. Although FUBAR is useful for identifying overall patterns of natural selection at amino acid encoding sites, it could not be used to analyze differential selection pressures on *M. tuberculosis* within HIV-1 coinfected and HIV-1 uninfected individuals. The reason for this is that to estimate the rates of synonymous and nonsynonymous substitutions, the FUBAR method derives information about the evolutionary history of the sequences being analyzed from the present time back till their most recent common ancestor: a history which in our case would have been largely shared between *M. tuberculosis* strains sampled from HIV-1 coinfected and uninfected individuals.

In order to differentiate between selection acting on *M. tuberculosis* strains within HIV-1 uninfected and HIV-1 coinfected individuals it was necessary to apply a selection analysis approach such as that implemented in the MEDS method (Murrell et al. 2012). The MEDS method identifies signals of directional selection associated with a specific environmental variable (in this case HIV-1 coinfection). To do this sequences are labelled as being on either the “foreground” or “background” branches of a phylogenetic tree based on the value of an associated binary variable, such as, in our case, the HIV-1 status of the patient from which the sequences were sampled. The foreground/background designations provide a frame of reference indicating the directionality (from background to foreground) of the selection that is to be assessed. The MEDS method then identifies codon sites as being directionally selected when these incur nonsynonymous substitutions that preferentially map to phylogenetic tree branches that separate the background branches from the foreground branches. Previous studies have suggested that in settings where both *M. tuberculosis* and HIV prevalence are high, the polarity of *M. tuberculosis* transmission is primarily from HIV uninfected individuals to HIV coinfected individuals (Middelkoop et al. 2015; Eldholm et al. 2016). Therefore, for the MEDS analysis phylogenetic tree branches leading to *M. tuberculosis* sequences from HIV-1 coinfected individuals were labeled as being in the foreground, and all other branches of the phylogentic tree were labeled as being in the background. To approximate the probability that potentially directionally selected sites occurred more frequently in particular gene categories than could be accounted for by chance we devised a permutation test where the HIV-1 status of the analyzed *M. tuberculosis* genomes were randomly shuffled amongst the *M. tuberculosis* sequences. Altogether 100 separate permutated data sets were analyzed with MEDS as above. For a particular site category (such as essential gene, nonessential gene or epitope), the proportion of permuted data sets where the same number or more directionally selected sites was observed than was observed in the real data set represented the approximate probability of the observed number of directionally selected sites occurring in that site category by chance alone.

As an additional control, MEDS was run with *M. tuberculosis* sequences from HIV-1 uninfected individuals in the foreground and those from HIV-1 coinfected individuals in the background. Results of the MEDS and FUBAR analyses were filtered to exclude sites that occur in regions (Coscolla et al. 2015) for which short-read sequencing data might be unreliable (supplementary table 4, Supplementary Material online). MESQUITE v3.04 (Maddison and Maddison 2017) was used to visualize the mappings of individual substitutions at particular codon-sites in the *M. tuberculosis* phylogeny. The SLAC (Kosakovsky Pond and Frost 2005) method implemented in HYPHY was used to determine the inferred number of amino acid changes at each site.

### Functional Categorization of FUBAR and MEDS Results

Each codon site analyzed by FUBAR or MEDS was categorized into essential, nonessential, or epitope site categories based on data described in (Sassetti et al. 2003). To define epitope-encoding regions, a list of *M. tuberculosis* epitopes experimentally confirmed to interact with CD4^+^ and CD8^+^ human T-cells was downloaded from the International Epitope Database (IEDB) (Vita et al. 2015) on February 26, 2015. Genome coordinates for each epitope were obtained by a BLAST analysis of the linear sequence of each epitope against the H37Rv reference genome.

### *Mycobacterium* *t* *uberculosis* *–*Human–HIV-1 Interaction Network

Possible biological factors underlying signals of HIV-1 coinfection associated directional selection in specific *M. tuberculosis* genes were investigated by finding the shortest pathways with the highest weighting between these genes and HIV-1 genes along a currently unpublished functional *M. tuberculosis*–human–HIV-1 protein interaction network. The network was constructed by combining existing intra-species human and *M. tuberculosis* functional protein interaction networks, as well as interactions between human and *M. tuberculosis* genes and human and HIV-1 genes. The intraspecies human and *M. tuberculosis* functional interaction networks were generated according to the methods of Rapanoel et al. (2013) with the exclusion of interactions inferred from sequence data and using more recent data from STRING (Szklarczyk et al. 2015) and REACTOME (Croft et al. 2014; Fabregat et al. 2016). In STRING, interactions are assigned confidence scores which are derived by benchmarking the predicted interactions against KEGG (Kanehisa et al. 2016), a trusted source of functional associations. The intraspecies interactions were only included if the confidence score was greater than 0.7: a score that is considered to indicate high confidence (von Mering et al. 2005). The human–HIV-1 interactions were derived from the HIV-1 Human Protein Interaction Database (Fu et al. 2009) by filtering on interactions recorded in at least two publications and with interaction types in one of the following categories as defined in (MacPherson et al. 2010): modification, degradation, physical or binding. The human–*M. tuberculosis* interactions were derived from previously generated data sets (Rapanoel et al. 2013; Huo et al. 2015). However for the purpose of this analysis, we only included human–*M. tuberculosis* interactions that had been reported in the literature. This large network will be the subject of a different publication. Here, we focused only on the small number of network edges and nodes that linked three particular *M. tuberculosis* genes to HIV-1 genes. The intraspecies edges in the network were weighted by their interaction score, and the human–HIV-1 edges were weighted by the number of publications they were reported in. The shortest paths from each of the three *M. tuberculosis* genes to the HIV-1 genes were calculated using NetworkX (Schult and Swart 2008). Thereafter, the sum of the weights along all the edges in the paths was calculated to determine the highest weighted path between each *M. tuberculosis* gene and each HIV-1 gene in the network.

## Supplementary Material

Supplementary data are available at *Molecular Biology and Evolution* online.

## Supplementary Material

Supplementary Data
