# Supplementary material for: The Influence of HIV on the Evolution of Mycobacterium tuberculosis
Source: Mol Biol Evol. 2017 Mar 21;34(7):1654–68. doi: 10.1093/molbev/msx107 (PMC5455964; doi:10.1093/molbev/msx107)
Supplement: Supplementary Data [file msx107_Supp.zip › SUPP_FIGURES_MBE-16-0989.R1_Koch et al_The influence of HIV on the evolution of Mtb.pdf]

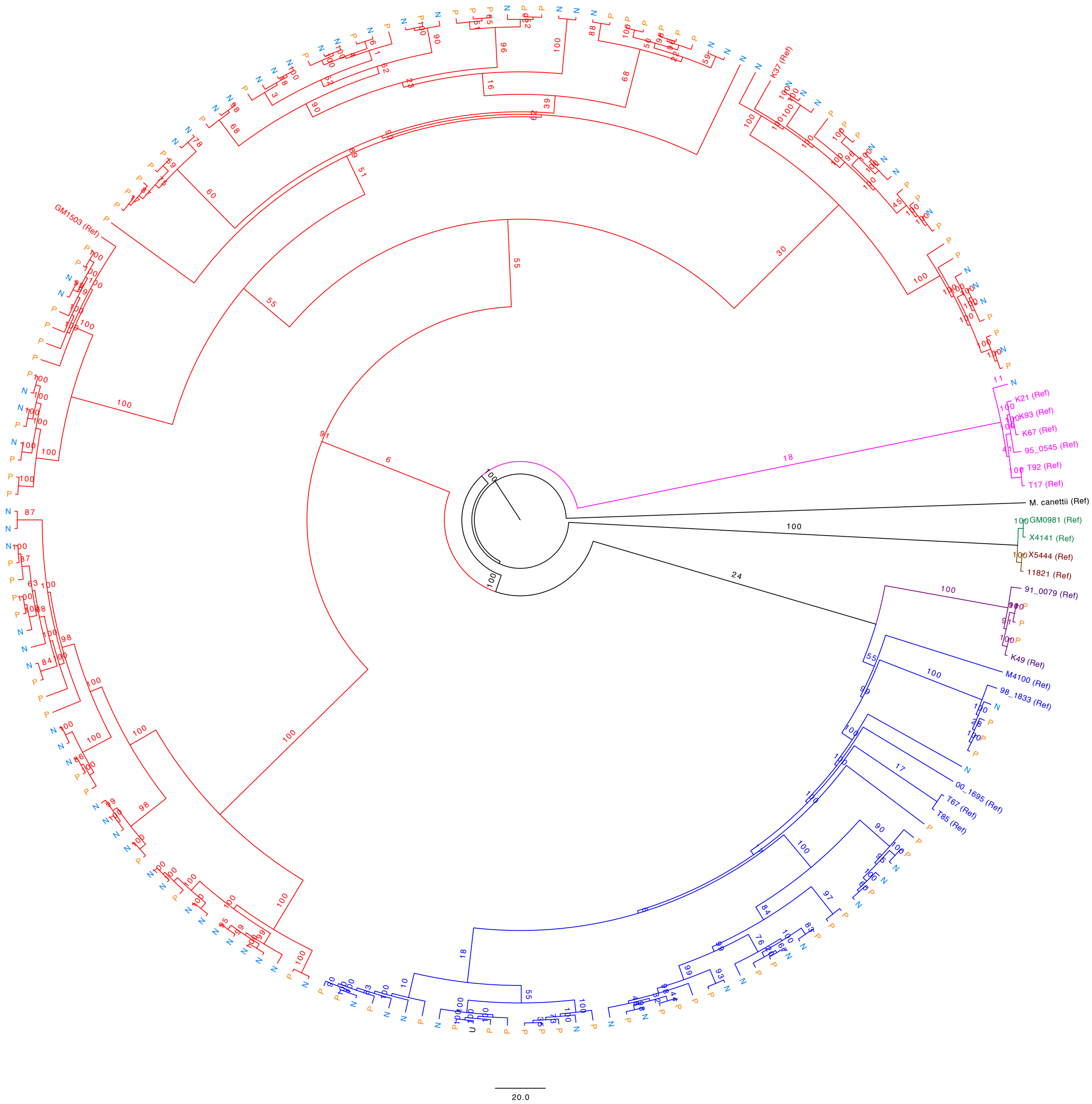

Supplementary figure 1. Phylogeny of *M. tuberculosis* strains isolated from HIV-1 co-infected and HIV-1 uninfected individuals with associated bootstrap values. Maximum likelihood phylogenetic tree, constructed using RAxML (Stamatakis 2014), is shown here with bootstrap values from 1000 replicates indicated on the branches. The reference set of strains, representative of the known global diversity of Mtb (Comas et al. 2010), are annotated as "Ref" on the tree. As in Figure 1 in the main text, *M. canettii* is included as an outgroup and blue and red branches indicate strains belonging to lineage 2 and 4, respectively. Other lineage branches are coloured as in (Comas et al. 2010). Strains isolated from HIV-1 uninfected individuals (N) are indicated in light blue, and those from HIV-1 co-infected individuals (P) in orange.

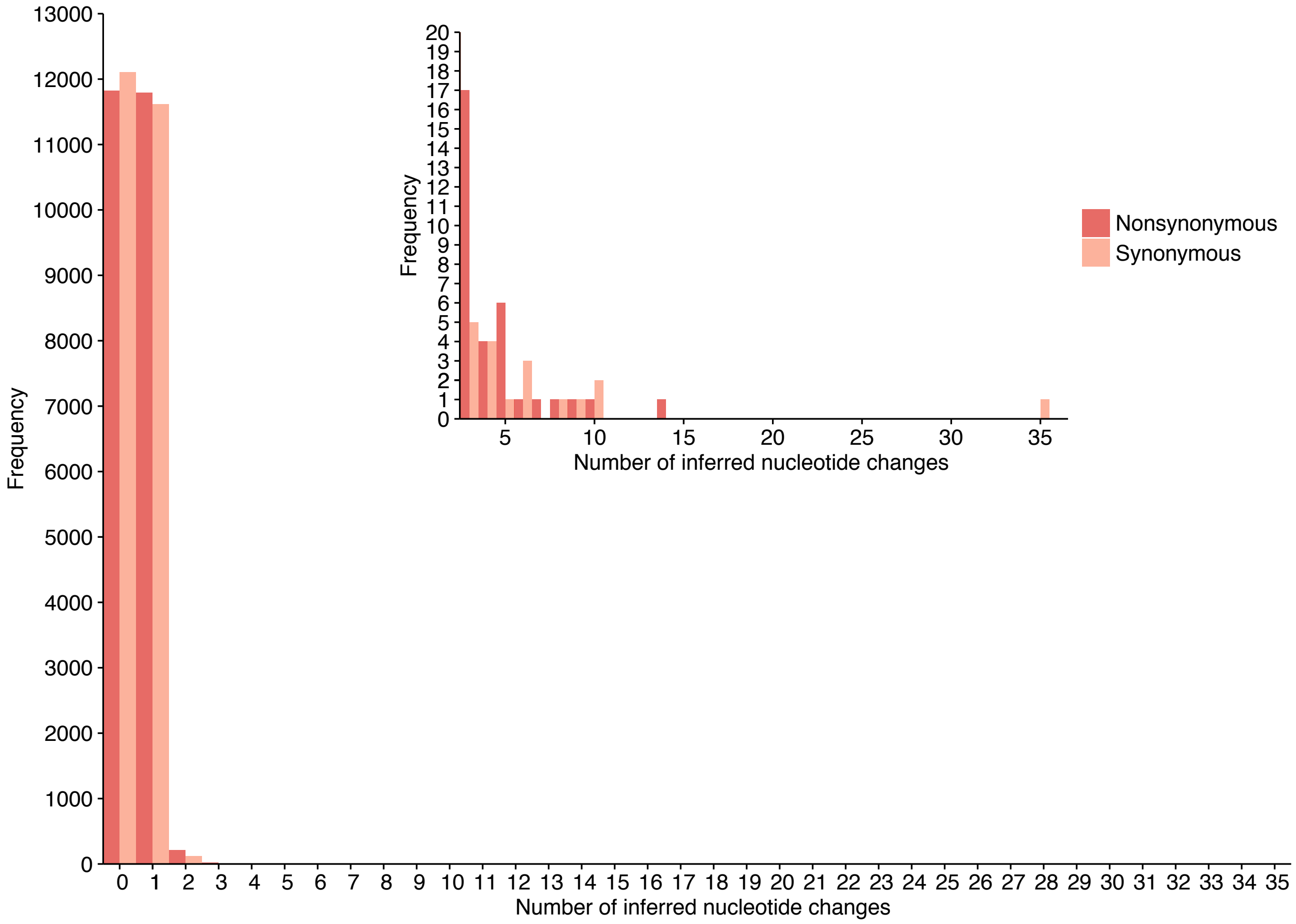

Supplementary figure 2. Distribution of the inferred number of synonymous and nonsynonymous mutations. The number of inferred synonymous and nonsynonymous nucleotide changes across the phylogeny was determined using SLAC (Kosakovsky Pond and Frost, 2005) implemented in HYPHY. The main graph shows the distribution of these values for all codon sites. The large majority of sites have between 0 and 2 inferred substitutions, thus the distribution is heavily skewed. The inset graph shows the distribution of the data for sites that have between 3 and 25 inferred amino acid changes.

A

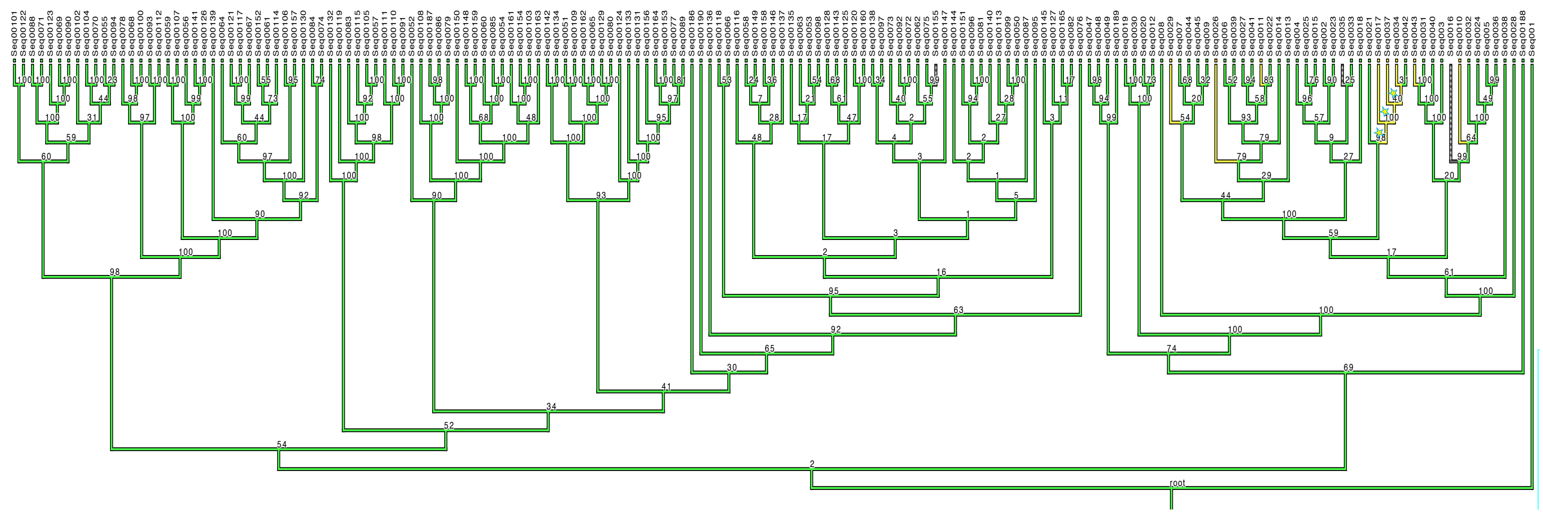

Rv0988, L191V, C517G, dN-dS = 19.40, 6 strains

B

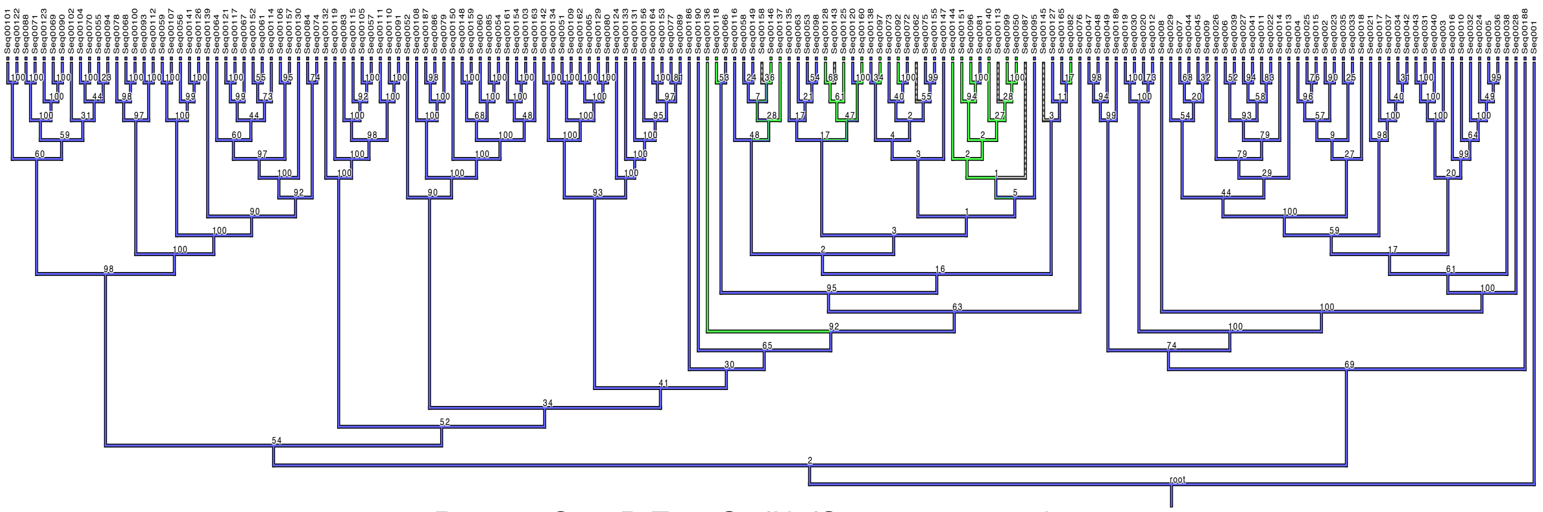

Rv1417, S102P, T304C, dN-dS = 10.01,16 strains

C

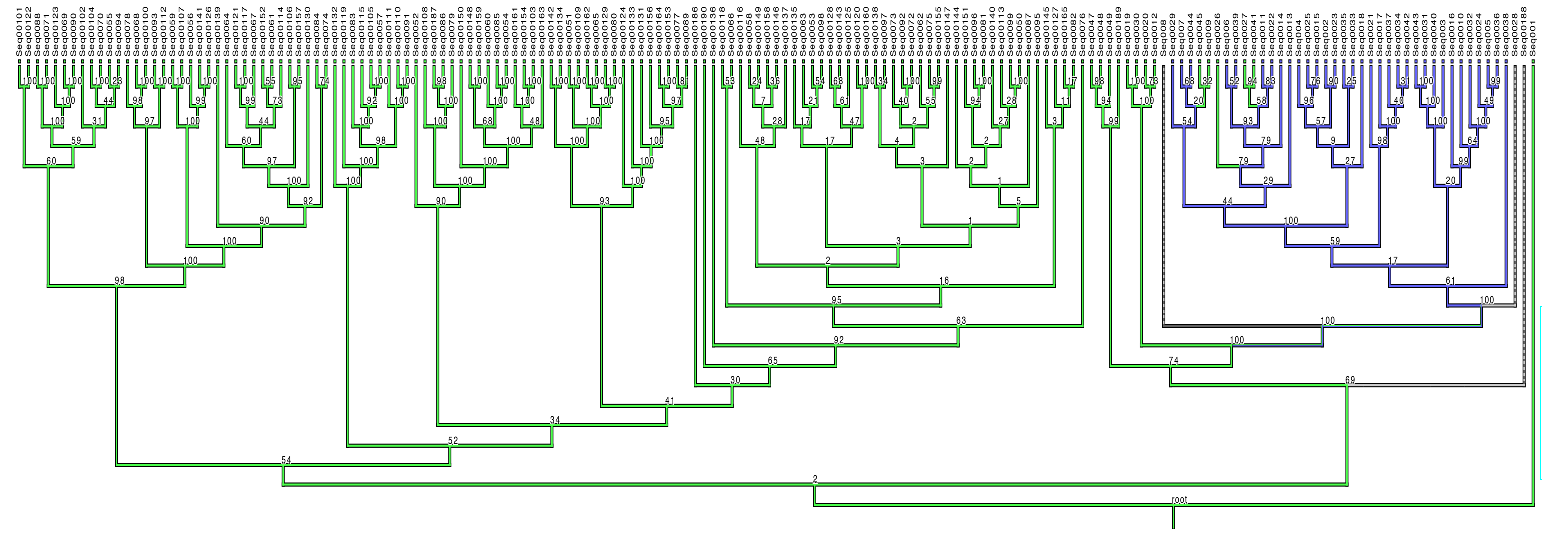

Rv0136, P114S, C340T, dN-dS = 8.99, 33 strains

Supplementary figure 3. Phylogenetic mapping of SNPs under the strongest degree of positive selection according to FUBAR. The phylogenetic position of codon sites with the highest dN-dS values reported by FUBAR (Murrell et al., 2013) were mapped using MESQUITE (Maddison and Maddison, 2001). RAxML (Stamatakis 2014) was used to generate phylogenetic trees and bootstrap values from 1000 replicates are annotated on nodes. The gene in which the codon occurs, the amino acid change, the nucleotide change, the FUBAR reported dN-dS value and the percentage of strains that have this mutation are indicated under each tree.

A

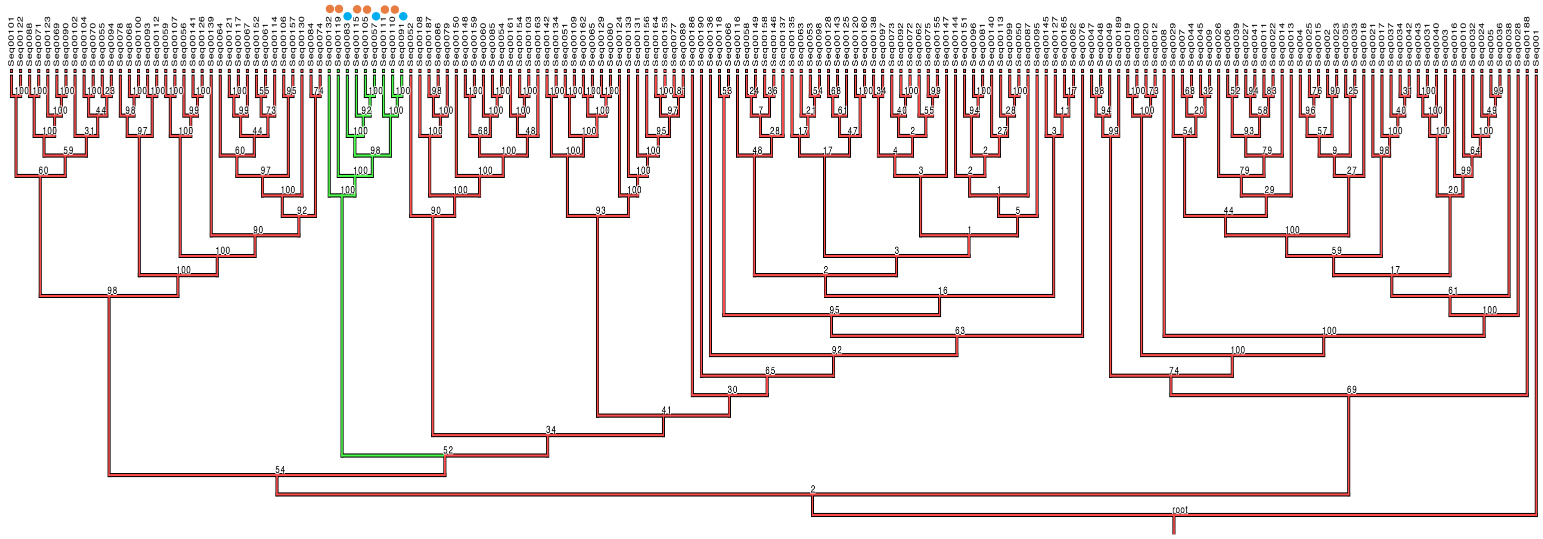

Mutation with median p value for evidence of differential selection in strains isolated from HIV-1 infected individuals (p = 0.0319)
Mutation: Rv1520, K217Q, A649C
Total no. strains with mutation: 9, no. strains from HIV-1 infected individuals with mutation: 6

B

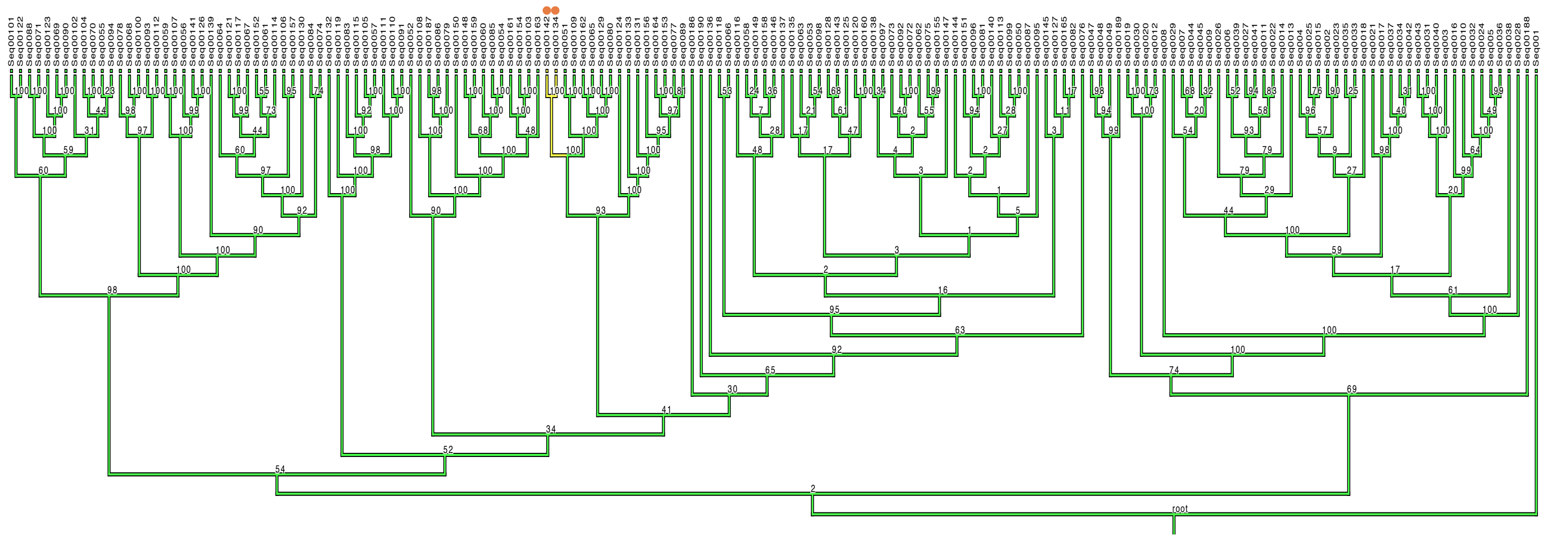

Mutation with weakest evidence of differential selection in strains isolated from HIV-1 infected individuals (p = 0.0499)
Mutation: Rv3699, P21A, C61G
Total no. strains with mutation: 2, No. strains from HIV-1 infected individuals with mutation: 2

Supplementary figure 4. Phylogenetic mapping of Mtb codon sites with evidence for HIV-1 co-infection associated episodic directional selection. MESQUITE (Maddison and Maddison, 2001) was applied to map the phylogenetic position of codon sites representative of different MEDS associated p-values. For each site the mutation, the degree of p-value support, the total number of strains containing the mutation and the number of strains from HIV-1 co-infected individual is indicated. RAxML (Stamatakis 2014) was used to generate phylogenenies and bootstrap values from 1000 replicates are indicated on the nodes. *M. tuberculosis* strains with the particular mutation isolated from HIV-1 co-infected individuals are indicated with an orange dot, and those from HIV-1 uninfected individuals a blue dot. **A)** Phylogenetic mapping of a codon site with MEDS associated p-value support of 0.032 is indicated. This substitution (K217Q in Rv1520) involves a single nucleotide substitution from adenine (in red) to cytosine (in green). **B)** The codon site with the lowest p-value in this dataset (p = 0.049) occurs in Rv3699 and involves a P21A substitution. The nucleotide change at this site is from a cytosine (in green) to a
